# Supplementary material for: Content-rich biological network constructed by mining PubMed abstracts
Source: BMC Bioinformatics. 2004 Oct 8;5:147. doi: 10.1186/1471-2105-5-147 (PMC528731; doi:10.1186/1471-2105-5-147)
Supplement: Additional File 2 — The original results of the above study (non-essential files are deleted to keep the file size under the limit set by BMC bioinformatics). [file 1471-2105-5-147-S2.bz2 › chilibotAdditionalFile2/dip05/434ID7622448E10379/html/CCL4_CCR3.html]

 


 **CCL4** and **CCR3** 
  
Found 13 abstracts in PubMed, retrieved 05.  
 

 What does Google say? 
 PDF only 
| .edu only 

---

- Eur J Immunol, 2002   **Gene copy number regulates the production of the human chemokine CCL3 L1.** .
  Genetic variation in the chemokine system is likely to affect responses to infection, and influence the course of autoimmune and inflammatory disease.
  We and others have shown that the human beta chemokine CCL3 L1, unlike its related non allelic isoform CCL3, has high affinity for the chemokine receptors D6, **CCR3** and CCR5.
  Moreover, CCL3 L1, but not CCL3, is susceptible to cleavage by CD26, creating a truncated 2 form with enhanced affinity for CCR1 and CCR5.
  Strong interaction with CCR5 means that CCL3 L1, and particularly its 2 variant, are by far the most potent natural HIV entry inhibitors described to date.
  Here, using real time PCR we have shown that CCL3 L1 and a novel **CCL4** isoform termed **CCL4** L1 can vary from 1 6 copies per diploid genome pdg in Caucasians and are occasionally completely absent.
  The other isoforms CCL3 and **CCL4** remain at two copies per dpg.
  Importantly, in a model system of pro inflammatory chemokine production LPS activated monocytes higher gene copy number correlates with an increased ratio of CCL3 L1 versus CCL3 mRNA, and enhanced chemokine production.
  Supernatants from samples with high copy number are able to more potently chemoattract CCR5 expressing cells, an effect blocked with anti CCL3 CCL3 L1 antibodies.
  As a result of these studies, we hypothesize that genetic variation in CCL3 L1 gene copy number may affect the susceptibility to, or the progression or severity of, diseases in which this chemokine plays a role.

  - J Neurol Neurosurg Psychiatry, 2002   **Expression of chemokines in the CSF and correlation with clinical disease activity in patients with multiple sclerosis.** .
    OBJECTIVE To define the chemokine profile in the CSF of patients with multiple sclerosis MS and compare it with three control groups.
    patients with benign headache headache , non inflammatory neurological diseases NIND , and other inflammatory neurological diseases IND .
    In addition, the correlations of CSF chemokine concentrations with chemokine receptor expression on CSF CD4 T cells and with clinical disease activity were assessed.
    METHODS Forty three patients with MS, 24 with IND, 44 with NIND, and 12 with benign headache undergoing diagnostic or therapeutic lumbar puncture were included.
    Supernatant fluid from CSF was analysed for four beta CCL2, CCL3, **CCL4**, CCL5 and two alpha CXCL9, CXCL10 chemokines by enzyme linked immunosorbent assay ELISA .
    Chemokine receptors **CCR3**, CCR5, and CXCR3 on CD4 T cells from eight patients with MS were analysed using directly conjugated fluorescent labelled monoclonal antibodies and flow cytometry.
    RESULTS CXCL10, formerly interferon gamma inducible protein 10 IP 10 , was significantly increased and CCL2, formerly monocyte chemoattractant protein 1 MCP 1 , was significantly reduced in the CSF of patients with MS and IND compared with those with benign headache and NIND.
    Concentrations of CXCL10 were significantly greater in patients with relapsing remitting compared with secondary progressive MS and correlated significantly with CXCR3 expression on CSF CD4 T cells from patients with MS.
    Concentrations of CXCL10 decreased and CCL2 concentrations increased as time from the last relapse increased in patients with MS.
    CONCLUSION Increased CXCL10 and decreased CCL2 concentrations in the CSF are associated with relapses in MS.
    Although serial values from individual patients were not available, this study suggests that CXCL10 and CCL2 may return towards baseline concentrations after a relapse.
    Correlation of CXCL10 with CD4 T cell expression of CXCR3 was consistent with its chemoattractant role for activated lymphocytes.
    Thus CXCL10 neutralising agents and CXCR3 receptor antagonists may be therapeutic targets in MS.

    - Virology, 2002   **Chemokine induction and leukocyte trafficking to the lungs during murine gammaherpesvirus 68 MHV 68 infection.** .
      Murine gammaherpesvirus 68 replicates in the alveolar epithelium and induces an inflammatory infiltrate in the lung, following intranasal challenge, and is cleared 10 and 13 days after infection by a T cell dependent mechanism.
      In order to understand the development of the immune response to this virus and how leukocyte trafficking to the lung is regulated, chemokine expression during MHV 68 infection was examined in lung tissue using an RNase protection assay.
      Expression of RANTES, eotaxin, MIP 1 alpha, MIP 1 beta **[CCL4]**, IP 10, and MCP 1 was upregulated by day 7 after infection.
      Chemokine concentrations in lung lavage fluid were also determined by ELISA.
      MCP 1, RANTES, MIP 1 alpha, eotaxin, and KC were upregulated during MHV 68 infection.
      Most of these chemokines have been reported to be chemoattractants for either activated T cells or monocytes, which are the major cellular components of the inflammatory infiltrate induced by the virus.
      Upregulated expression of the corresponding receptors for the chemokines, including CCR1, CCR2, **CCR3**, CCR5, and CXCR3, coincided with the development of the inflammatory infiltrate.
      The chemokine levels peaked at around day 7 after infection, coinciding with peak viral titers and slightly preceding maximal T cell infiltration.
      In vitro chemotaxis assays confirmed that lung lavage fluid from MHV 68 infected mice had chemotactic activity, which was partially blocked by antibodies to IP 10 and RANTES.
      These observations suggest that the chemokines detected play an important role in regulating leukocyte trafficking to the lungs during MHV 68 infection.

      - J Neuroimmunol, 2001   **Beta CC chemokines as modulators of sleep implications for HIV induced alterations in arousal state.** .
        Sleep is altered early in the course of HIV infection, before the onset of AIDS, indicating effects of the virus on neural processes.
        Previous observations suggest HIV envelope glycoproteins are possible mediators of these responses.
        Because some beta CC chemokine receptors serve as co receptors for HIV and bind HIV envelope glycoproteins, we determined in this study whether selected CC chemokine ligands alter sleep and whether their mRNAs are detectable in brain regions important for sleep.
        **CCL4** MIP 1beta, but not CCL5 RANTES, injected centrally into rats prior to dark onset increased non rapid eye movements sleep, fragmented sleep, and induced fever.
        mRNA for the chemokine receptor **CCR3** was detectable under basal conditions in multiple brain regions.
        These data suggest some CC chemokines may also be involved in processes by which HIV alters sleep.

        - Blood, 2001   **Eotaxin is a natural antagonist for CCR2 and an agonist for CCR5.** .
          Eotaxin is a potent inducer of eosinophil chemotaxis and was considered as a selective ligand of the CC chemokine receptor 3 **[CCR3]** **CCR3** , which is expressed on eosinophils, basophils, and Th2 lymphocytes.
          This study shows that eotaxin also interacts with CCR2 and CCR5 and can, thus, affect the responses of monocytes, which express both receptors.
          In human monocytes pretreatment with eotaxin decreased responsiveness to MCP 1, a selective ligand for CCR2, as well as to RANTES and MIP 1 beta **[CCL4]**, which bind to CCR5.
          Similar effects were obtained with transfected cells expressing CCR2 or CCR5, but here a difference became apparent Eotaxin triggered CCR5 at a concentration of 100 nM but not CCR2 even at 1 microM, suggesting an antagonistic effect on this receptor.
          In agreement with this observation, eotaxin induced internalization of CCR5 but not of CCR2 in human monocytes and transfected cells.
          Binding studies showed that eotaxin displaces 125 I MCP 1 from monocytes in a concentration dependent manner, and functional experiments showed that eotaxin inhibits MCP 1 induced chemotaxis and enzyme release.
          The results demonstrate that eotaxin is a CCR5 agonist and a CCR2 antagonist.
          The present findings suggest a role of eotaxin in the fine tuning of cellular responses occurring at sites of allergic inflammation, in which both MCP 1 and eotaxin are produced.
          Blood.
          2001;97 1920 1924
